# Targeting the metabolic profile of amino acids to identify the key metabolic characteristics in cerebral palsy

**DOI:** 10.3389/fnmol.2023.1237745

**Published:** 2023-08-17

**Authors:** Dan Wang, Juan Song, Ye Cheng, Yiran Xu, Lili Song, Yimeng Qiao, Bingbing Li, Lei Xia, Ming Li, Jin Zhang, Yu Su, Ting Wang, Jian Ding, Xiaoyang Wang, Sujuan Wang, Changlian Zhu, Qinghe Xing

**Affiliations:** ^1^Children’s Hospital of Fudan University and Institutes of Biomedical Sciences of Fudan University, Shanghai, China; ^2^Henan Key Laboratory of Child Brain Injury and Henan Pediatric Clinical Research Center, Department of Pediatrics, The 3rd Affiliated Hospital and Institute of Neuroscience, Zhengzhou University, Zhengzhou, China; ^3^Obstetrics and Gynecology Hospital, Fudan University, Shanghai, China; ^4^Centre of Perinatal Medicine and Health, Sahlgrenska Academy, University of Gothenburg, Gothenburg, Sweden; ^5^Center for Brain Repair and Rehabilitation, Institute of Neuroscience and Physiology, University of Gothenburg, Gothenburg, Sweden; ^6^Shanghai Center for Women and Children’s Health, Shanghai, China

**Keywords:** cerebral palsy, amino acid metabolism, targeted quantification, preterm birth, diagnosis, biomarker, tryptophan, β-amino-isobutyric acid

## Abstract

**Background:**

Cerebral palsy (CP) is a neurodevelopmental disorder characterized by motor impairment. In this study, we aimed to describe the characteristics of amino acids (AA) in the plasma of children with CP and identify AA that could play a potential role in the auxiliary diagnosis and treatment of CP.

**Methods:**

Using high performance liquid chromatography, we performed metabolomics analysis of AA in plasma from 62 CP children and 60 healthy controls. Univariate and multivariate analyses were then applied to characterize different AA. AA markers associated with CP were then identified by machine learning based on the Lasso regression model for the validation of intra-sample interactions. Next, we calculated a discriminant formula and generated a receiver operating characteristic (ROC) curve based on the marker combination in the discriminant diagnostic model.

**Results:**

A total of 33 AA were detected in the plasma of CP children and controls. Compared with controls, 5, 7, and 10 different AA were identified in total participants, premature infants, and full-term infants, respectively. Of these, β-amino-isobutyric acid [*p* = 2.9*10^(−4)^, Fold change (FC) = 0.76, Variable importance of protection (VIP) = 1.75], tryptophan [*p* = 5.4*10^(−4)^, FC = 0.87, VIP = 2.22], and asparagine [*p* = 3.6*10^(−3)^, FC = 0.82, VIP = 1.64], were significantly lower in the three groups of CP patients than that in controls. The combination of β-amino-isobutyric acid, tryptophan, and taurine, provided high levels of diagnostic classification and risk prediction efficacy for preterm children with an area under the curve (AUC) value of 0.8741 [95% confidence interval (CI): 0.7322–1.000]. The discriminant diagnostic formula for preterm infant with CP based on the potential marker combination was defined by *p* = 1/(1 + e^−(8.295–0.3848* BAIBA-0.1120*Trp + 0.0108*Tau)^).

**Conclusion:**

Full-spectrum analysis of amino acid metabolomics revealed a distinct profile in CP, including reductions in the levels of β-amino-isobutyric acid, tryptophan, and taurine. Our findings shed new light on the pathogenesis and diagnosis of premature infants with CP.

## Introduction

Cerebral palsy (CP) refers to a non-progressive motor dysfunction and postural syndrome caused by early brain damage and developmental defects with a global incidence of 2–3.5/1,000 live births (Little, 1966; Pharoah et al., 1998; Yeargin-Allsopp et al., 2008; Kruse et al., 2009; Himmelmann et al., 2010; Van Naarden et al., 2016). The etiology of CP is multifactorial and heterogeneous, and events in the prenatal, perinatal, and postnatal periods, alone or in combination, may lead to CP (Pakula et al., 2009; Sadowska et al., 2020). Previous studies have reported that 80% of all CP cases are caused by fetal brain damage induced by adverse prenatal events, including intrauterine growth retardation, intrauterine hypoxia, preterm delivery, multiple pregnancies, prenatal bleeding, and intrauterine infectious (Nelson, 2008; Sun et al., 2008; Wu et al., 2013; MacLennan et al., 2015). Preterm birth is a major causative factor for CP; furthermore, the risk increases with reducing gestational age (Spittle et al., 2018). Children born preterm account for more than 30% of children with CP (Demesi et al., 2016; Korzeniewski et al., 2018).

CP refers to a variety of symptoms with strong heterogeneity. Typically, CP patients present with abnormalities on brain examination, electroencephalogram (EEG), and functional testing, although some cases do not fully meet the criteria for a definitive diagnosis (Korzeniewski et al., 2018). The current diagnostic basis of CP is based on clinical symptoms, a practice that can easily lead to subjective bias. Furthermore, CP can be confused with other forms of brain dysplasia or diseases with a similar phenotype. During early developmental stages, children may not be diagnosed in a timely manner due to the inconspicuous or non-typical symptoms. Consequently, these children miss the opportunity for early treatment or rehabilitation, thus leading to a poorer prognosis and outcome. Therefore, there is an urgent need to identify specific biomarkers for CP that can provide basic evidence to facilitate early screening, auxiliary diagnosis, and targeted therapeutics.

The full spectrum amino acids (AA) remain in a state of dynamic equilibrium in the physiological state, and any imbalance may induce a disease state (Wu, 2009; Wu, 2010; Shivakumar et al., 2018). Imbalances may also be secondary to original diseases, particular diseases involving metabolism, nervous system, cardio-cerebrovascular system, tumors, and senility (Semba et al., 2016; Riedl et al., 2017). In contrast to previous metabolomics studies, which tended to focus on proteins and AA, the full spectrum analysis of AA can detect and evaluate the body and the balance of amino acid metabolism from a more comprehensive and systematic perspective. The identification of abnormal amino acid metabolism could provide valuable clues to enhance our understanding of pathogenesis, diagnostic molecular markers, and potential clinical intervention.

AA are important neurotransmitters, which serve as chemical messengers playing a crucial role in information processing of central neurons (Wu, 2009; Wu, 2010). Previous studies revealed a significant association between neurodevelopmental disorders and abnormalities in the amino acid spectrum (Semba et al., 2016). Plasma levels of branched-chain amino acids (BCAAs) were found to be reduced in patients with autism, epilepsy, intellectual disability, and other neurodevelopmental disorders, due to the mutation and inactivation of the *BCKDK* gene (Novarino et al., 2012). The higher newborn cord BCAA levels are associated with a higher risk of developing attention-deficit hyperactivity disorder in childhood (Anand et al., 2021). Furthermore, *BCKDK* knockout mice presented with an abnormal amino acid distribution in the brain and neurobehavioral deficits (Novarino et al., 2012). Other researchers have reported crucial evidence to support the involvement of amino acid metabolism in the pathogenesis of Alzheimer’s disease (AD) (Griffin and Bradshaw, 2017; Kim et al., 2019), and have identified potential biomarkers for the diagnosis and treatment of autism (Smith et al., 2018). In addition, a dietary pattern with relatively low caloric intake from proteins may increase the risk of mild cognitive impairment (MCI) or dementia in elderly persons (Roberts et al., 2012). The chronic deficiency of AA can lead to impaired neurotransmission, resulting in cognitive and memory impairments, and direct brain supplementations of glutamine and BCAAs can effectively mitigate neurodegenerative outcomes as well as functional degeneration (Chow et al., 2021). However, although CP is a common neurodevelopmental disease, no previous study has investigated the metabolomics of AA in CP. In the present study, we report the metabolic characteristics of children with CP children by considering the full spectrum of AA and performing high flux targeted quantitative detection. We also provide an efficient predictive diagnostic formula and therapeutic possibilities for children with CP.

## Methods

### Participant enrollment

The study cohort featured 62 children with CP and 60 controls from the Third Affiliated Hospital of Zhengzhou University. All participants underwent a detailed clinical evaluation and comprehensive pre-test counseling. We collated a range of clinical information, including demographic variables such as sex, age, gestational age, and birth weight. This study protocol was approved by the Human Research Ethics Committee of Zhengzhou University (ethical number 201201002) and performed in accordance with the Helsinki Declaration. All participants or their legal representatives provided written and informed consent.

### Diagnosis, classification, and exclusion criteria

Clinical pediatric rehabilitation specialists confirmed the diagnosis of spastic CP cases using criteria related to non-progressive motor control and postural disorders. We excluded children with congenital craniocerebral malformation, metabolic encephalopathy, hereditary encephalopathy, and a family history of other neurological disorders (Rosenbaum et al., 2007). The control group featured healthy children without neurological disease who matched the demographic characteristics of the case group and were followed up in the same hospital.

### Sample processing and experimentation

Peripheral blood samples were collected in the morning according to the requirements of metabolomic sampling. After establishing the internal standard as 5% salicylic acid solution (including 100 μg/mL theanine) and the determining the pH of the extraction solution by performing preliminary experiments, we generated a mixed standard solution containing 43 types of free AA and 380 μg/mL of organic extract (0.1 mol/L HCL·ISTD, 10% TCA = 1:2). We added the same volume of organic extract to 50 μL of each plasma sample to remove protein, lipids, and pigments, and then added 10 μL of internal standard solution to adjust the pH value to 1.7–2.2. Next, the mixture was centrifuged for 20 min (16,688 g) at room temperature and the supernatant was drawn into an injection bottle with a lined tube, and then stored at 4°C. An amino acid analyzer [Hitachi L-8900 (Hitachi, Tokyo, Japan)] was then used to determine the levels of each amino acid, using the following pre-set parameters: 4.6 mm id × 60 mm cationic resin filling chromatographic column, 4°C injector temperature, 20 μL injection volume, 45°C column temperature, wavelengths of 570 and 440 nm dual channel for UV detection, a 3 pmol detection limit, six types of buffer, and 90 min of testing time. Nine quality control (QC) samples were used for all samples.

### Data analysis

According to Beale’s law, the retention time and peak area of the AA were analyzed qualitatively and quantitatively. Actual amino acid content was defined as systematic detection value × (plasma sample volume + extract volume)/extract volume. In order to evaluate the stability and accuracy of the experimental data, the relative standard deviation (RSD) of each group of data was obtained using the QC samples and internal standard values of each sample.

Statistical analysis of clinical characteristics, data cleaning, univariate analysis, machine learning, and discriminant analysis were carried out using R software v.3.5.4 (R Foundation for Statistical Computing, Vienna, Austria). Comparisons between groups were carried our using the chi-squared test, *t-*test or the Wilcoxon rank sum test according to the distribution of data. For the univariate analysis of amino acid group comparisons, the *p-*value (<0.05 was considered statistically significant) of metabolites was calculated by the Wilcoxon rank sum test. In terms of the variation amplitude of the difference metabolites, the fold change (FC) ratio was calculated by dividing the mean values of the two groups (CP group/control group). For multivariate analysis, we performed principal component analysis (PCA), partial least squares discriminant analysis (PLS-DA), and orthogonal partial least squares discriminant analysis (OPLS-DA) in Simca v.13.0 (UMETRICS AB, Umea, Vasterbotten, Sweden). In addition, 5-fold cross validation was performed by machine learning to identify differential AA and to obtain the receiver operating characteristic (ROC) curves and the area under the curve (AUC) values based on the Lasso regression model. The training set is the data sample used for model fitting, and validation set is a separate set of samples set aside during model training. In the cross validation, it randomly subdivided all the samples in the CP and control group into different training and validation sets to train the model, according to the proportion of 70 and 30%. A discriminant analysis formula was deduced for the diagnosis of disease based on the ROC curve for specific amino acid combinations based on the multiple logistic regression model.

## Results

### Clinical characteristics

We analyzed 62 patients and 60 controls and there 42 and 46 males, and 29 and 20 premature infants, in these groups, respectively. The diagnosis age of children in the CP group ranges from 9 to 36 month, with a mean of 20.97 ± 6.96 month. The patients and controls were well-matched in terms of gender, age, gestational age, and birth weight, thus indicating that our data was not biased with respect to these clinical parameters (Table 1).

**Table 1:** Clinical characteristics of the study subjects.

Category		Cerebral palsy group	Control group	*p*-value
Number		62	60	–
Gender	Male	42 (66.7%)	46 (76.7%)	0.27*
	Female	20 (32.3%)	14 (23.3%)	
Age (month)		20.97 ± 6.96	21.13 ± 7.29	0.92^#^
Gestational age (week)	Preterm	29 (46.8%)	20 (33.3%)	0.13* 0.22^#^
	Term	33 (53.2%)	40 (66.7%)	
		35.9 ± 4.2	37.0 ± 3.4	
Birth weight (g)		2691.3 ± 990.7	2836.7 ± 793.8	0.15^#^

* and # were calculated by chi-squared and *t*-tests.

### Chromatograms of amino acid composition

**Figure 1:**
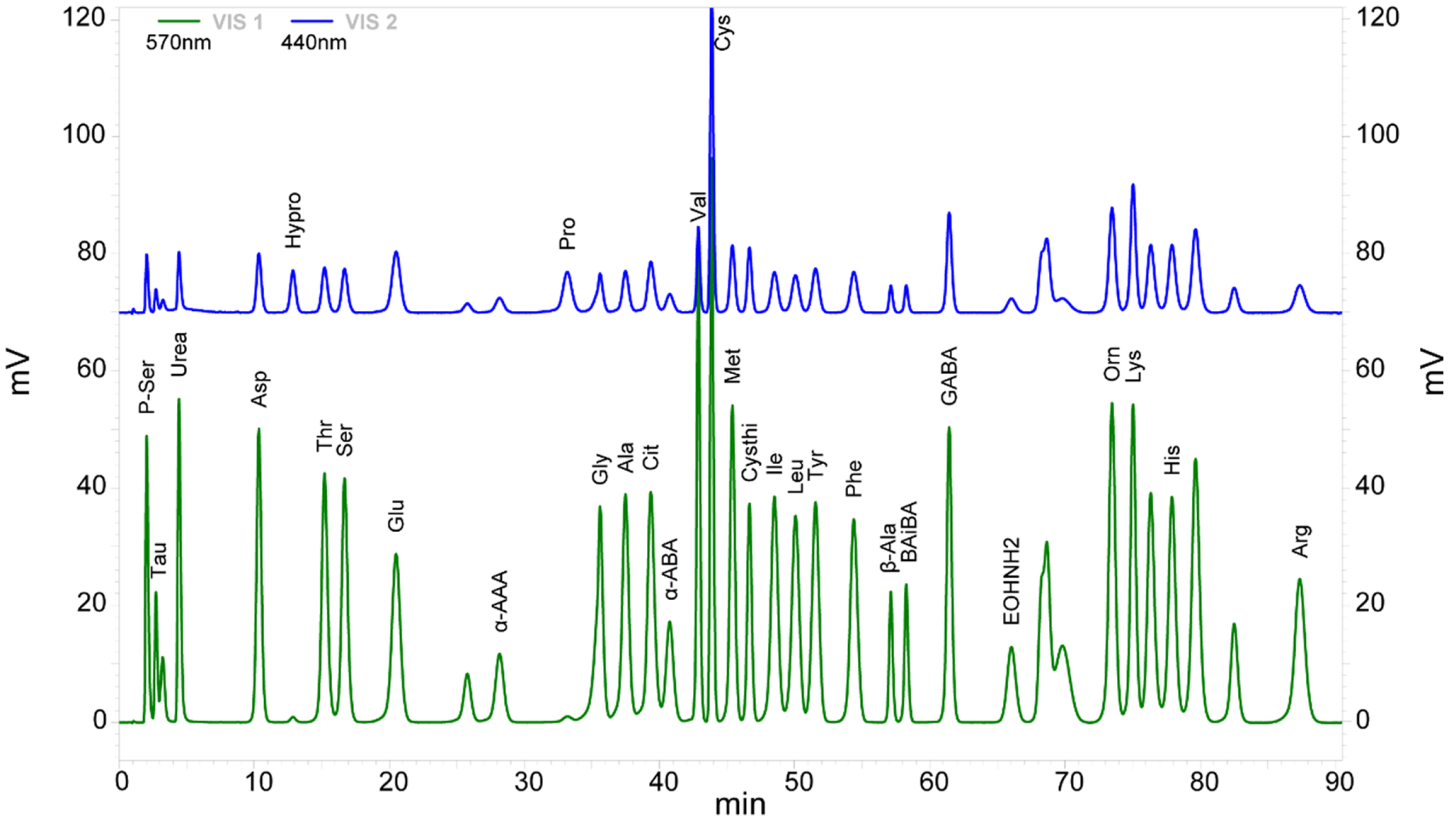
Chromatogram of 33 amino acid components detected in the cerebral palsy samples. VIS1 (green) and VIS2 (blue) are amino acid chromatograms of the maximum absorption of color reaction at 570 and 440 nm under ultraviolet light, respectively.

Thirty-three AA were detected in the plasma of CP children and controls. The chromatograms of each amino acid component (as shown in a single sample) showed a stable peak. No significant deviation was detected with respect to the peak for the contrast mixed standard (Figure 1).

### Data quality control assessment

The relative standard deviations of the peak area for each amino acid in nine QC samples were calculated and presented according to retention time. Of these, the mean peak area for the internal standard theanine was 29,363,836 ± 865,409.01, and the corresponding RSD was 2.95%. The RSD value for the 33 AA investigated was far less than the RSD < 30% requirement of clinical samples in metabolomics research (Supplementary Figure S1), thus indicating that our analysis was reliable and stable.

### Distribution of different AA

**Figure 2:**
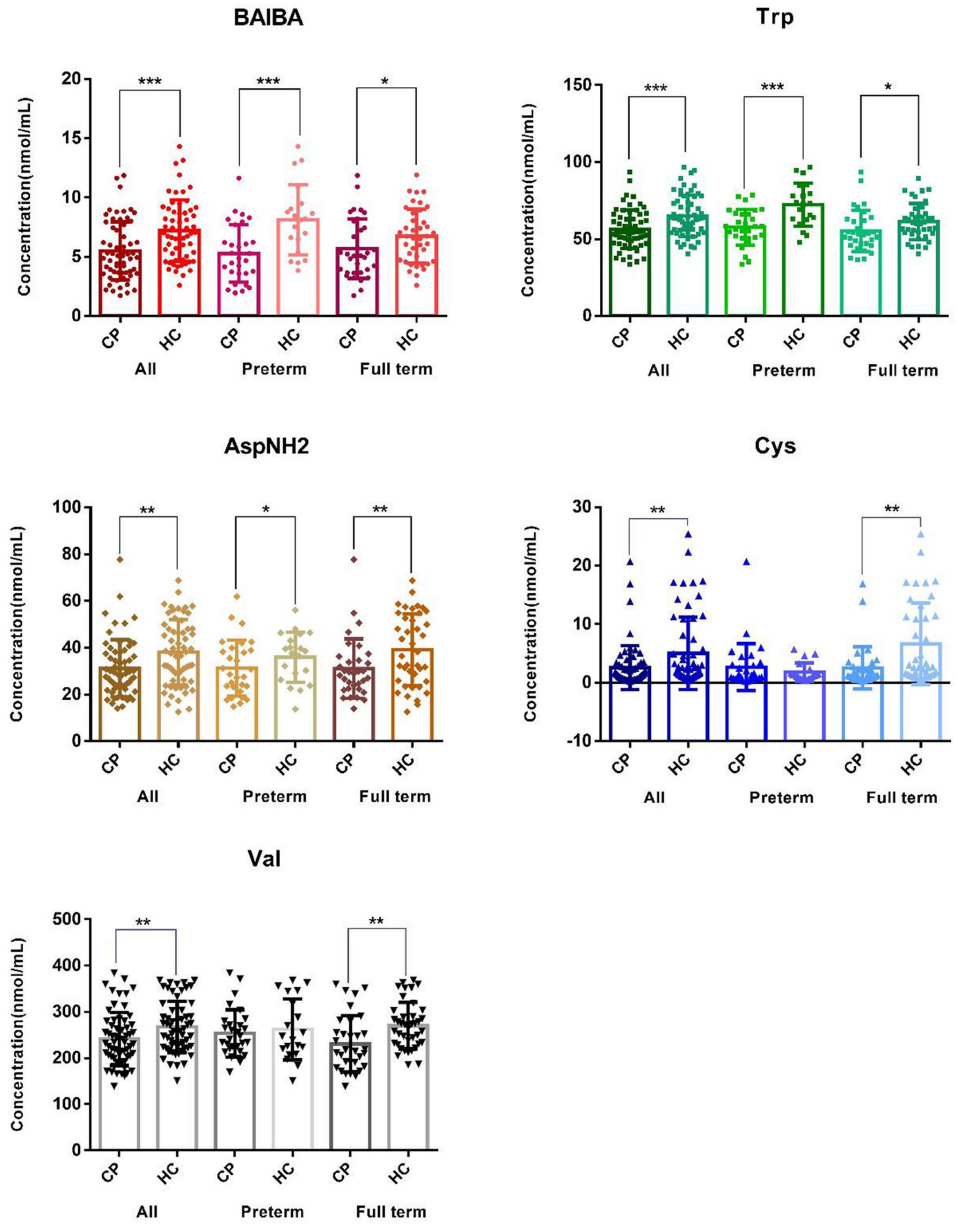
Distribution of the major amino acids showing differences between the cerebral palsy group and the control group. The graph of BAIBA, Trp, AspNH2, Cys, Val, and α-AAA shows differences in β-amino-isobutyric acid, tryptophan, asparagine, cystine, valine, and amino adipic acid between the total sample, the premature samples, and the full-term samples, respectively. CP and HC represent the cerebral palsy group and the healthy control group, respectively. ALL, Preterm and Full term represent all subjects, premature infants, and full-term infants, respectively. ***p < 0.001, **p < 0.01, and *p < 0.05.

In this targeted quantitative full spectrum detection of AA, the 33 AA were subjected to univariable statistical analysis in order of retention time (small to large; Supplementary Table S1). Globally, the plasma concentrations of five AA were significantly lower in the CP group than in the control group, including β-amino-isobutyric acid (BAIBA) with the most significant difference [*p* = 2.9*10^(−4)^], tryptophan (Trp), asparagine (AspNH2), cystine (Cys) with the largest FC of 0.52, and valine (Val) (Figure 2 and Supplementary Table S1).

**Figure 3:**
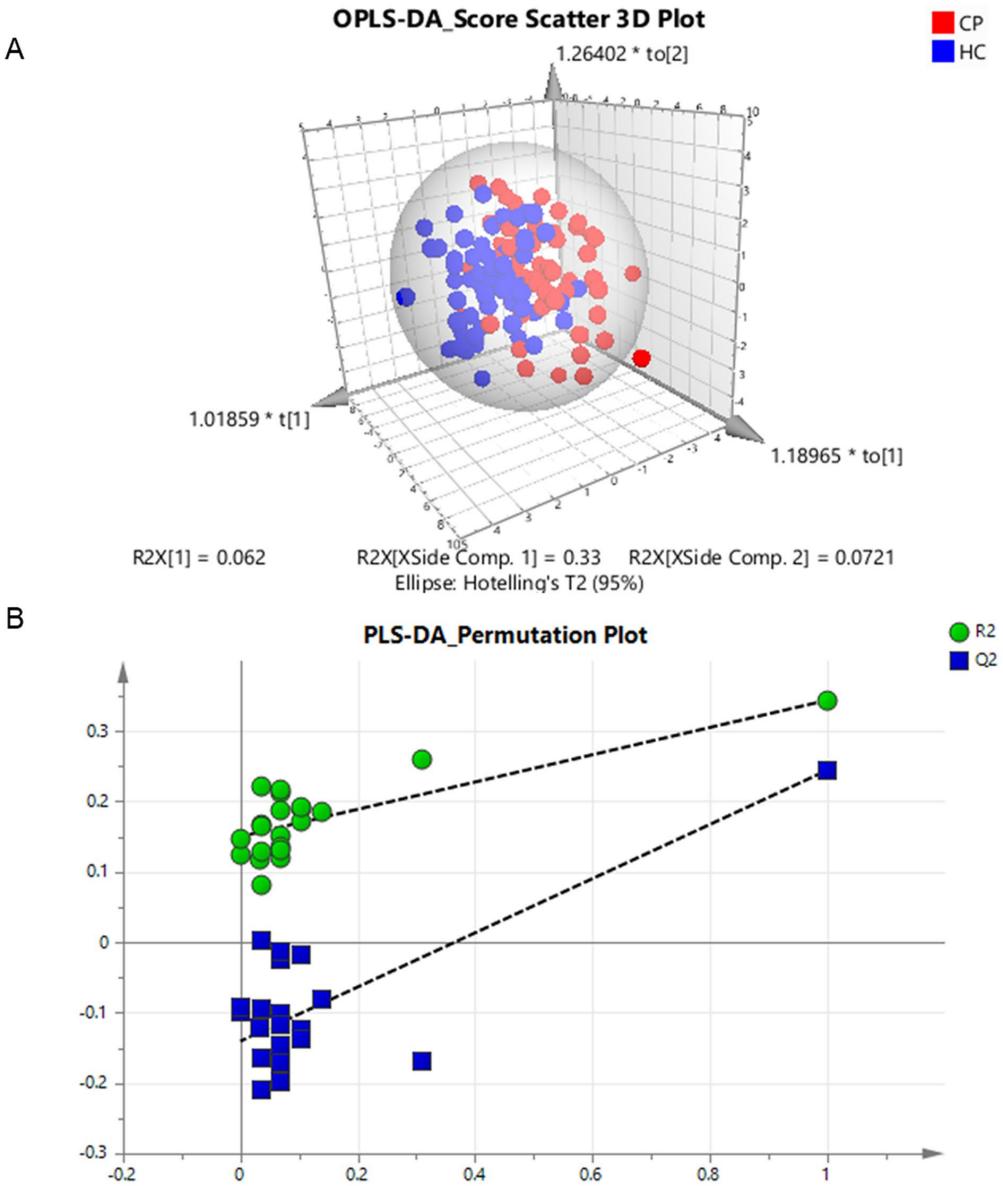
Multivariate analysis of differential amino acids in the children with cerebral palsy. **(A)** Component differences between cerebral palsy and control groups in the OPLS-DA classification prediction model of human amino acids, CP and HC represent the cerebral palsy and the control group, respectively. **(B)** PLS-DA model 1,000 permutation test results, the X-axis designates the correlation coefficient between original and permuted response data, and the Y-axis represents R^2^Y and Q^2^Y for every model. R^2^ and Q^2^ reflect the goodness of fit for the model.

Subgroup analysis of premature children (29 cases vs. 20 controls) identified seven AA that were significantly different (*p* < 0.05), including β-amino-isobutyric acid, tryptophan, amino adipic acid (α-AAA), phenylalanine (Phe), arginine (Arg), taurine (Tau) and asparagine. Of these, the differences for β-amino-isobutyric acid [*p* = 5.6*10^(−4)^] and tryptophan [*p* = 6.6*10^(−4)^] remained significant after Bonferroni correction (Figure 2 and Supplementary Table S1). In the full term group, we identified 10 AA that showed significant differences between the 33 cases and 40 controls (*p* < 0.05), including cystine with the most significant difference [*p* = 2.2*10^(−3)^], valine, asparagine, citrulline, tryptophan, urinary sulfur ether (Cysthi), leucine (Leu), serine (Ser), α-aminobutyric acid (α-ABA) and β-amino-isobutyric acid (Figure 3 and Supplementary Table S1). However, the level of significance for each of these AA disappeared after Bonferroni correction for multiple testing.

### Multivariate analysis

After removing six outliers located outside the 95% confidence interval ellipse in the multivariate analysis of PCA, we generated an OPLS-DA classification prediction model. The discriminant analysis of the two groups in supervised mode revealed a clear distinction, with the CP group at the right side of ellipsoid and the control group on the left side. The model corresponds to 1 + 2 principal components; the R^2^X was 0.464 (0.062 + 0.33 + 0.0721), the R^2^Y was 0.461, and the Q^2^ was 0.202 (Figure 3A).

After 20-fold randomly shuffled sample grouping, the PLS-DA model was evaluated by the replacement test. All the blue Q^2^ values on the left were lower than the initial values on the right, and the intercept of the blue regression line at the point Q^2^ was −0.14 (< 0). Furthermore, the slope of the line was greater than 0 and R^2^ was 0.15, thus indicating the efficacy of the original model without any fitting (Figure 3B).

In the multivariate analysis at the overall level, we also identified five significantly different AA; their contribution to the classification of the groups (from highest to lowest) were tryptophan (VIP = 2.22), β-amino-isobutyric acid (VIP = 1.87), cystine (VIP = 1.80), valine (VIP = 1.75), and asparagine (VIP = 1.64); these findings were consistent with those provided by univariate analysis.

### Machine learning

The ROC curve for single AA did not demonstrate sufficient discrimination. Thus, the machine learning method was used to further screen amino acid markers for CP. In the process of feature reduction in the Lasso regression model, tryptophan, cystine, and serine were captured and the corresponding model was found to be the most stable with the lowest variance (Supplementary Figures S2A, C). The independent ROC curves for each marker corresponded to the AUC values of tryptophan (0.6792), cysteine (0.6437), and serine (0.5057), thus indicating that the classification efficacy of individual markers was poor (Supplementary Figure S2B). In this model, the AUC value of the ROC curve corresponding to the training set after the combination of markers was 0.7799, and the AUC value for the validation set after internal cross-validation was 0.7367 with a 95% confidence interval (CI) of 0.6104–0.8630 (Supplementary Figure S2C). After screening, we determined the combination of AA with certain diagnostic efficacy in the CP group and the control group.

**Figure 4:**
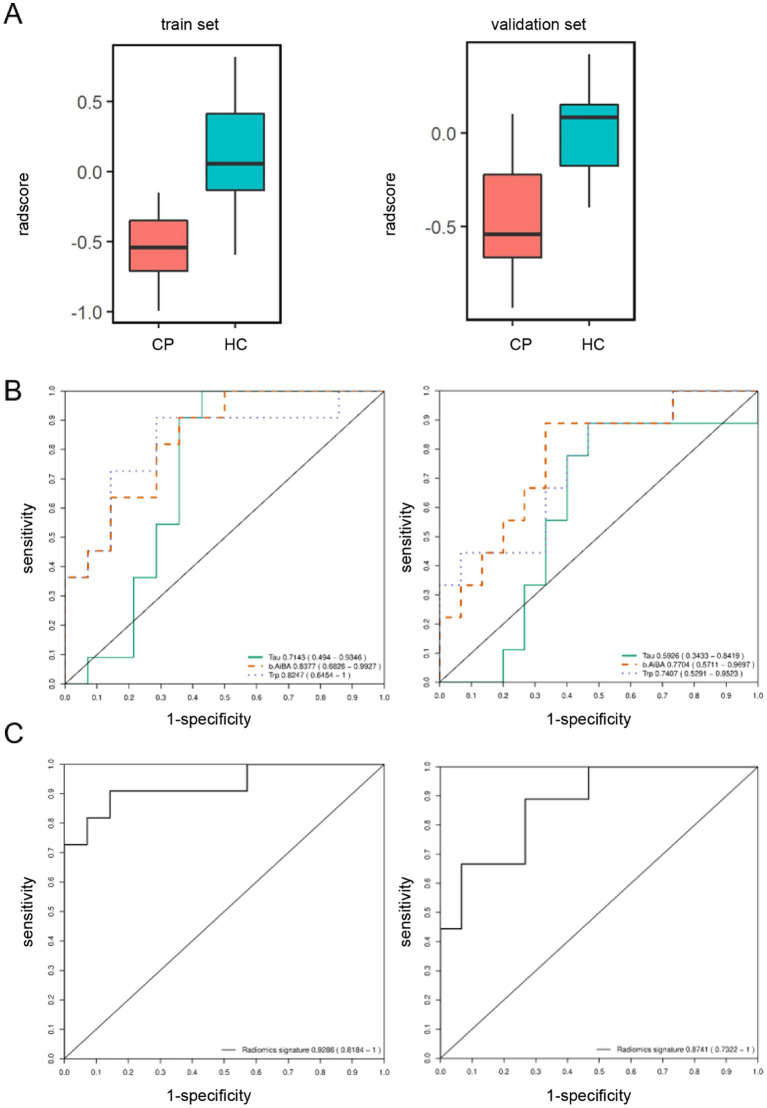
Screen for amino acid markers associated with cerebral palsy in preterm children by machine learning. **(A)** Box chart of marker combination scores in the model. Red and blue box represent the cerebral palsy and the control group. The train set and validation set consisted of 70% and 30% samples which were randomly divided according to two groups of 122 samples, respectively. **(B)** ROC curve for each primary differential marker in the model. Tau (green solidline curve), BAIBA (orange dotted curve) and Trp (purple dotted curve) represent the discrimination situation of taurine, β-amino-isobutyric acid and tryptophan. **(C)** ROC curve for the primary differential marker combination in the model.

In addition, we also conducted the cross-validation and marker screening in the CP groups and control groups for premature and mature infants. In the premature samples, the selected markers included in the combination set were β-amino-isobutyric acid, tryptophan, and taurine. The corresponding AUC values for the three ROC curves in the validation set were 0.7704, 0.7407, and 0.5926, respectively, thus indicating poor classification efficacy (Figure 4B). In the model, the ROC corresponding to the AUC values in the biomarker group were 0.9286 for the training set and 0.8741 for the validation set with a 95% CI of 0.7322–1.0000 (Figure 4C). The classification efficacy of the combination was significantly improved, thus confirming that the two sets were stable (Figure 4A). We also identified a combination of AA with high diagnostic potency for premature CP children and control children by applying machine learning screening. However, the AUC of the validation for the combination of cysteine, cystithione, and proline in term infants was 0.7152 with a 95% CI of 0.5476–0.8827, thus indicating low diagnostic efficacy (Supplementary Figure S3).

### Generation of a discriminant diagnostic model

**Figure 5:**
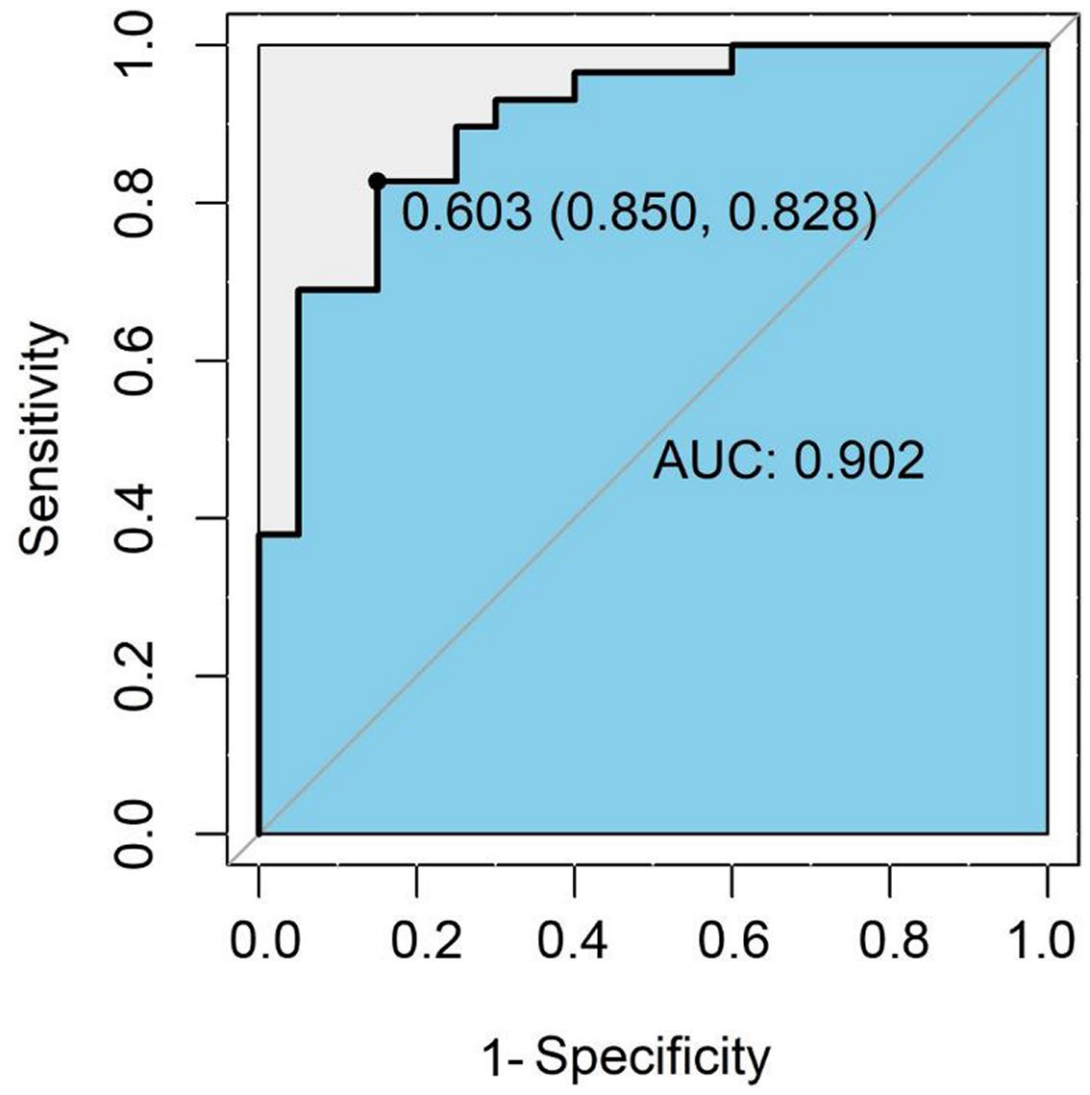
ROC curve for the amino acid combinations used for discriminative diagnosis. The ROC curve is a coordinate graph composed of 1-specificity (false positive rate) on the horizontal axis and sensitivity (true positive rate) on the vertical axis. The larger the area under the curve (blue area), the higher the diagnostic accuracy of the combination of β-amino-isobutyric acid, tryptophan and taurine.

The combination of β-aminoisobutyric acid, tryptophan, and taurine in plasma samples can be used as a potential marker combination for the auxiliary diagnosis of preterm birth CP. In practical application, preterm infants with CP can be recognized and diagnosed according to the following formula *p* = 1/(1 + e^-(8.295–0.3848* BAIBA-0.1120*Trp + 0.0108*Tau)^), as derived from the results of multiple logistic regression with β-aminoisobutyric acid, tryptophan, and taurine. BAIBA, Trp, and Tau represent the concentrations of β-aminoisobutyric acid, tryptophan, and taurine in the plasma of preterm infants (nmol/mL), respectively. When P is ≥0.603, the model indicates that the preterm infant is a child with CP; when P is <0.603, the model indicates that the preterm infant is not a CP child. The concentration threshold for discriminant diagnosis was the threshold corresponding to the maximum AUC (0.902) of the ROC curve for the combination of the three AA with a specificity of 0.850 and a sensitivity of 0.828 (Figure 5); this discriminant was more effective than a single amino acid (Supplementary Figure S4).

In summary, by deploying different analysis methods (univariate, multivariate analysis and machine learning), we determined that the levels of tryptophan and β-amino-isobutyric acid were reliably different when compared between the CP group and the control group (Table 2).

**Table 2:** Different amino acids screened by three analytical methods.

Analytical method	Differential amino acids
Univariate statistical analysis	All: β-amino-isobutyric acid, tryptophan, asparagine, cystine, valine
	Preterm: β-amino-isobutyric acid, tryptophan, amino adipic acid, phenylalanine, arginine, taurine, asparagine
	Full-term: cystine, valine, asparagine, citrulline, tryptophan, urinary sulfur ether, leucine, serine, α-aminobutyric acid, β-amino-isobutyric acid
Multivariate statistical analysis	All: tryptophan, β-amino-isobutyric acid, cystine, valine, asparagine
Machine learning	All: tryptophan, cystine, serine
	Preterm: β-amino-isobutyric acid, tryptophan, taurine
	Full-term: cysteine, cystithione, proline

## Discussion

This study was the first to systematically examine the distribution of full spectrum AA in plasma of children with CP, revealing the correlation between the full spectrum AA and CP. The detection of a metabolic imbalance of AA associated with neural function in CP children provides new evidence support a role of AA in the pathogenesis of CP.

In the current study, we detected a down-regulation of tryptophan in the plasma of CP children and validated this finding by a diverse range of analytical methods. As an essential amino acid, more than 95% of free tryptophan is degraded through the kynurenine pathway; its metabolite, kynurenine (Kyn), is known to play an important role in the regulation of neurofunction, immune response and intestinal homeostasis (Maddison and Giorgini, 2015; Muneer, 2020). Previous studies have reported that tryptophan metabolism represents a therapeutic target for neurodegenerative disorders (Platten et al., 2019). In addition, tryptophan is known to be involved in protein synthesis and the production of neurotransmitters, such as 5-hydroxytryptamine (5-HT) (Le Floc’H et al., 2011; Stone et al., 2013; van der Goot and Nollen, 2013) to promote the transduction of neuronal signals. In the brain, tryptophan, Kyn and 3-hydroxykynurenine (3HK) are taken up by astrocytes, microglia and neurons after being transported across the blood–brain barrier. Tryptophan is further converted into 5-HT and Kyn. Astrocytes mainly produce the neuroprotective kynurenic acid (KA) using Kyn as substrate, while microglia produce the neurotoxic quinolinic acid (QA) from 3HK (Platten et al., 2019); the observed reduction of tryptophan in CP patients may be related to the accumulation of QA produced by Kyn from tryptophan metabolism. In addition, QA may be converted to NAD+ in cells, a coenzyme that is critical for energy metabolism, an important aspect of embryonic development.

Tryptophan is known to alleviate chewing impairments observed in children with CP by increasing serotonergic activity, further broadening the important role of tryptophan in neurophysiological regulation (Lacerda et al., 2019). In addition, experimental studies have highlighted the potential of therapeutic nutritional strategies in attenuating the deficits of CP children. Given that there are no specific drugs for the treatment of CP, the efficiency of non-early rehabilitation is limited. Our research provides further evidence supporting clinical investigations into determining whether therapeutic nutritional strategies could be used to attenuate the functional and morphological damage created by CP. A deficiency of tryptophan and serotonin in the brain arising from excessive dieting and food restriction may lead to a number of neuropsychiatric disorders, including neuro-anorexia, depression, psychosis, and hyperactivity disorder (Haleem, 2017). Our current results imply that the onset of CP may be related to the imbalance of amino acid metabolism *in vivo*, and that the supplementation of compound AA may represent a potential strategy to improve the prognosis of children with CP.

In this study, the most significant difference between CP children and controls was the amino acid β-amino-isobutyric acid, which was also reduced in the plasma of CP children. This is a non-protein amino acid that is a catabolite of thymine and the antiretroviral thymine analogs azidothymidine (AZT) and stavudine (d4T) (Begriche et al., 2010). As a muscle contraction-induced actin produced and secreted by skeletal muscles, the plasma concentration of β-amino-isobutyric acid increases with added physical activities (Molfino et al., 2017); this is consistent with the clinical feature of motor dysfunction in children with CP. Previous research also identified the correlation between physical activity and reduced levels of β-amino-isobutyric acid in muscle and plasma (Molfino et al., 2019).

In addition, β-amino-isobutyric acid was shown to enhance fatty acid oxidation and reduce the body weight of experimental mice by increasing the amount of leptin produced by white adipose tissue (Begriche et al., 2010); furthermore, a reduction of β-amino-isobutyric acid *in vivo* will reduce fatty acid oxidation and lead to an imbalance in energy homeostasis. According to our results, the levels of β-amino-isobutyric acid in CP children aged 1–3 years were low, implying that it is unable to exert the protective effect on these children with motor dysfunction. Thus, like other components of actin proteins (Das et al., 2020), β-amino-isobutyric acid may serve as a potential prognostic biomarker that reflects systemic metabolism and may also represent an attractive therapeutic target for the treatment of muscle and metabolic diseases.

Taurine is one of the most abundant free AA in the developing central nervous system (Huxtable, 1989; Benitez-Diaz et al., 2003). Alterations to taurine homeostasis can impact a number of biological processes, such as development of the central nervous system and the retina, osmolarity control, glucose regulation, calcium modulation, and inhibitory neurotransmission, anti-oxidant activity, membrane stabilization, reproduction, and immunity (El, 2019), and have been reported in neurodevelopmental, neurodegenerative and metabolic disorders (Wharton et al., 2004; El, 2019; Rafiee et al., 2022). Structurally similar to the neurotransmitter γ-aminobutyric acid (GABA) and glycine (Tochitani, 2017), extracellular taurine inhibits neuronal firing through the interaction with both GABA and glycine receptors (Oja et al., 1990; Oja and Saransaari, 2017). Given the possible cytoprotective actions of taurine, it is speculated that the increased concentration of taurine in the plasma of preterm children with CP might constitute a compensatory mechanism that attempts to alleviate the abnormal motor function (Rafiee et al., 2022).

Valine serves as substrate to involve the glutamate synthesis during synaptic activity in cultured cerebellar neurons (Wu, 2009; Bak et al., 2012). As a BCAA essential for neurodevelopment and neurodegeneration, valine metabolism plays an important role in neurological disorders such as AD (Xiong et al., 2022). Lower plasma or serum valine levels are correlated with accelerated cognitive decline, such as MCI and AD, and conversely, an increase in valine concentration is associated with reduced risk of AD (Polis and Samson, 2020; Xiong et al., 2022). The level of valine was decreased in the CP group, and dietary supplementation with BCAAs can improve neurological phenotypes in rodents and humans (Novarino et al., 2012), collectively suggesting that exogenous increase of valine intake may reduce the risk of CP or improve the outcome of affected individuals. Therefore, for pregnant women, premature infants, and CP children, nutritional therapy through exogenous supplementation of neuroprotective AA or mixtures and optimization of dietary to increase the intake of high-quality protein substances (Kurpad et al., 2006), may play a role in the management of CP.

Preterm birth is the leading cause of childhood mortality in children under 5 years-of-age, thus accounting for approximately 11% of all cases (Boyle et al., 2017). Premature infants are at risk of a number of health complications, especially CP, as they are less developed, less tolerant, and their brains are more sensitive to risk factors and more susceptible to damage (O’Shea, 2008). By applying a machine learning screening method, we identified the clear utility of amino acid biomarkers for distinguishing CP children from controls. The combination of β-amino-isobutyric acid, tryptophan, and taurine, which are higher risk factors for CP in preterm infants, had the best classification power. This means that the combined amino acid discriminant model generated herein provides a quantitative tool for the auxiliary diagnosis and risk assessment of premature infants with CP. Moreover, plasma can be easily obtained clinically, and different AA can be detected simultaneously by a unified amino acid analysis platform, thus indicating the feasibility of using the discriminant diagnostic model in clinical scenarios.

## Conclusion

In the present study, we performed full spectrum amino acid metabolomics in CP children and controls with targeted quantitative detection for the first time. Collectively, our findings provide clues to explain the potential pathogenesis of CP from the perspective of amino acid imbalance and identified key biomarkers for CP. Of these, tryptophan may be involved in the pathogenesis of CP, thus providing evidence for the role as a target supplement for the prevention, treatment and rehabilitation of children with CP. Furthermore, the metabolic imbalance of β-amino-isobutyric acid may be related to the motor dysfunction of CP children and the accumulation of taurine may be a compensatory result. Nevertheless, additional studies are required to confirm whether the three diagnostic AA are just markers of spastic CP or they are in fact involved in pathogenesis. In view of the amino acid metabolism disorders in CP children, the combination model of relevant AA established by machine learning can provide a quantitative basis for the auxiliary diagnosis and risk assessment of CP. Further researches are required to determine whether these biomarkers could be used to increase early detection of difficult to diagnose CP cases. In addition to traditional drugs and rehabilitation therapies, complementary nutrition-based approach may be a therapeutic potential treatment for CP.

## Data availability statement

The raw data supporting the conclusions of this article will be made available by the authors, without undue reservation.

## Ethics statement

The studies involving humans were approved by the Human Research Ethics Committee of Zhengzhou University. The studies were conducted in accordance with the local legislation and institutional requirements. Written informed consent for participation in this study was provided by the participants’ legal guardians/next of kin.

## Author contributions

QX and CZ conceived and designed the study. JS, YX, BL, LX, ML, and XW recruited subjects and collated clinical information. DW and YQ performed all of the laboratory work with the assistance of LS, JZ, YS, TW, and JD. DW developed models, reviewed results, and provided guidance on the methods. DW performed all data and statistical analyses with the help of YC, and drafted the manuscript. QX and SW critically revised the manuscript for important intellectual content. All authors contributed and critically reviewed the final version of the manuscript, read and approved the final manuscript.

## Funding

This research was supported by the Shanghai Municipal Commission of Science and Technology Research Project (19JC1411002), the National Key Research and Development Program from the Ministry of Science and Technology of the People’s Republic of China (2021YFC2700800), the National Natural Science Foundation of China (31972880, 32170615, 31371274, U21A20347), the National Key Research and Development Plan for Stem Cell and Transformation Research (2017YFA0104202), the Collaborative Innovation Center Project Construction for Shanghai Women and Children’s Health, Swedish Governmental Grants to Scientists Working in Health Care (ALFGBG-965197, ALFGBG-966034), the Swedish Research Council (2018–02267, 2021–01950, 2022–01019), and the Brain Foundation (FO2022-0120).

## Conflict of interest

The authors declare that the research was conducted in the absence of any commercial or financial relationships that could be construed as a potential conflict of interest.

## Publisher’s note

All claims expressed in this article are solely those of the authors and do not necessarily represent those of their affiliated organizations, or those of the publisher, the editors and the reviewers. Any product that may be evaluated in this article, or claim that may be made by its manufacturer, is not guaranteed or endorsed by the publisher.
