# Supplementary material for: Targeting the metabolic profile of amino acids to identify the key metabolic characteristics in cerebral palsy
Source: Front Mol Neurosci. 2023 Aug 17;16:1237745. doi: 10.3389/fnmol.2023.1237745 (PMC10470834; doi:10.3389/fnmol.2023.1237745)
Supplement: Supplementary file 2 [file Image_1.pdf]

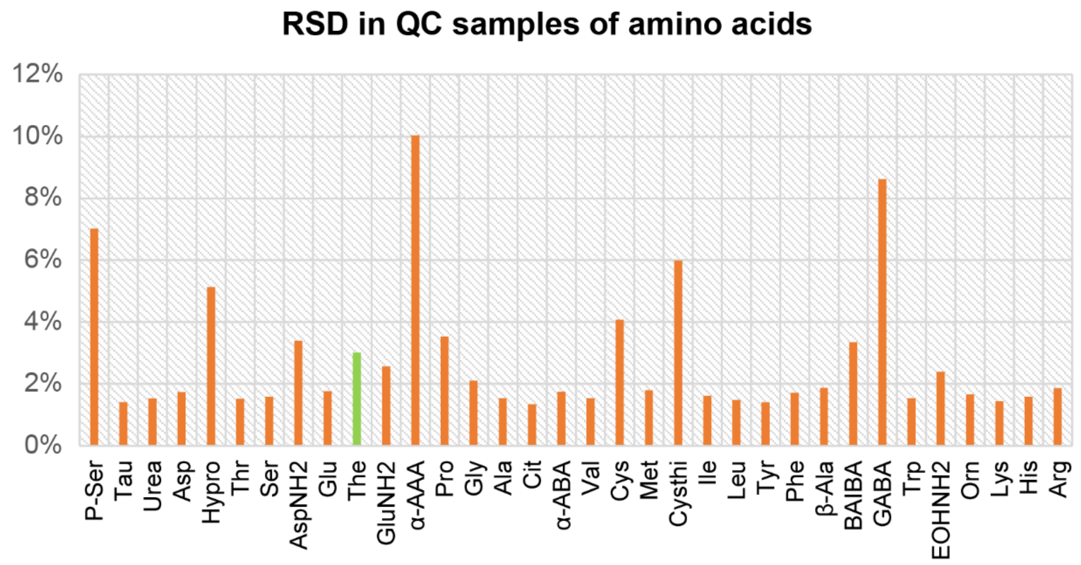

Supplementary Figure 1. Relative standard deviation of peak area of each amino acid in quality control

The orange columns represent the 33 amino acids detected in the sample, and the green columns represents the internal standard substance (theanine) added in the sample.
