# Supplementary material for: Targeting the metabolic profile of amino acids to identify the key metabolic characteristics in cerebral palsy
Source: Front Mol Neurosci. 2023 Aug 17;16:1237745. doi: 10.3389/fnmol.2023.1237745 (PMC10470834; doi:10.3389/fnmol.2023.1237745)
Supplement: Supplementary file 3 [file Image_2.pdf]

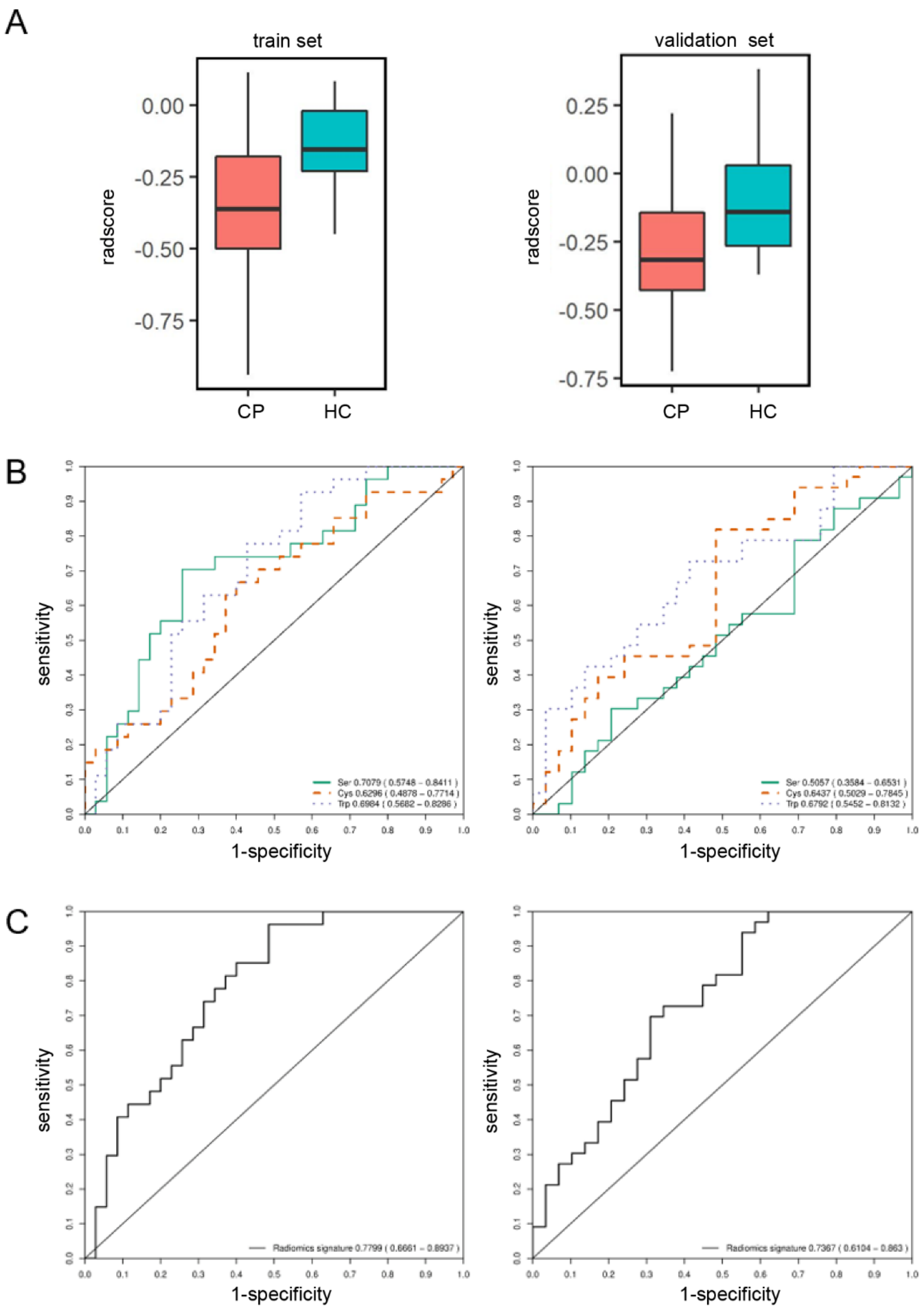

Supplementary Figure 2. Screen for amino acid markers associated with cerebral palsy in all study subjects by machine learning

The train set and validation set consist of 70% and 30% samples which are randomly divided according to the CP group and the control group of 122 samples respectively.

Figure A is box chart of marker combination' scores in the model. The red is the CP group and the blue is the control group.

Figure B is the ROC curve of each marker in the model.

Figure C is the ROC curve after the marker combination in the model.
