# Supplementary material for: Targeting the metabolic profile of amino acids to identify the key metabolic characteristics in cerebral palsy
Source: Front Mol Neurosci. 2023 Aug 17;16:1237745. doi: 10.3389/fnmol.2023.1237745 (PMC10470834; doi:10.3389/fnmol.2023.1237745)
Supplement: Supplementary file 5 [file Image_4.pdf]

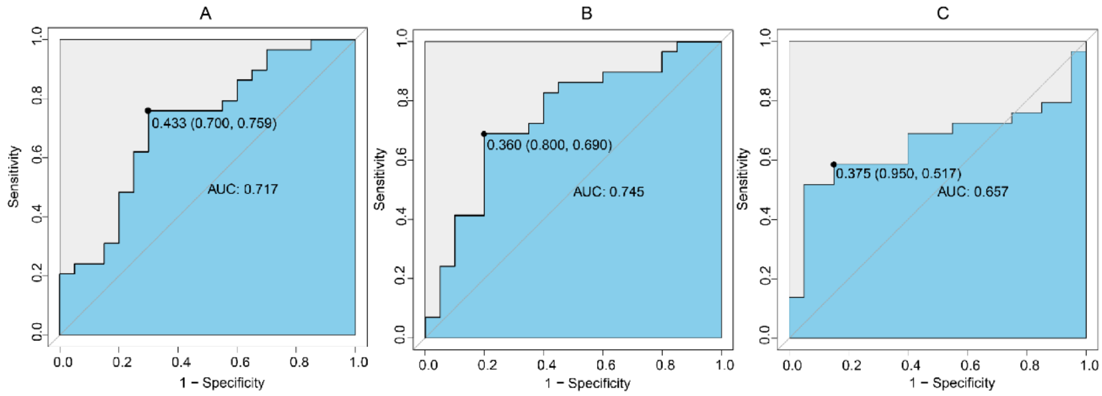

Supplementary Figure 4. ROC curve for single amino acids used for discriminative diagnosis

Receiver operating characteristic curve is a coordinate graph composed of 1-specificity (false positive rate) on the horizontal axis and sensitivity (true positive rate) on the vertical axis. The larger the area under the curve (blue area), the higher the diagnostic accuracy.

Figure A, B and C shows the ROC curves of β-aminoisobutyric acid, tryptophan, and taurine respectively.
